# Distinct Nuclear Organization of Telomeres and Centromeres in Monoclonal Gammopathy of Undetermined Significance and Multiple Myeloma

**DOI:** 10.3390/cells8070723

**Published:** 2019-07-15

**Authors:** Pak Lok Ivan Yu, Rachel R. Wang, Grace Johnston, Yaqiong Wang, Pille Tammur, Anu Tamm, Mari Punab, Aline Rangel-Pozzo, Sabine Mai

**Affiliations:** 1 Cell Biology, Research Institute of Oncology and Hematology, University of Manitoba, CancerCare Manitoba, Winnipeg, MB R3C 2B7, Canada; 2 Department of Botany and Zoology, University of British Columbia, Vancouver, BC V5Z 4E8, Canada; 3 Department of Microbiology, University of Manitoba, Winnipeg, MB R3T 2M6, Canada; 4 Department of Neuroscience, University of Winnipeg, Winnipeg, MB R3B 2E9, Canada; 5 Jilin University, Changchun 130021, China; 6 United Laboratories, Tartu University Hospital, 50406 Tartu, Estonia; 7 Department of Haematology and Bone Marrow Transplantation, Clinic of Haematology and Oncology, Tartu University Hospital, 50406 Tartu, Estonia

**Keywords:** multiple myeloma, monoclonal gammopathy of undetermined significance, telomeres, centromeres, structural genome markers, genomic instability

## Abstract

Both multiple myeloma (MM) and its precursor state of monoclonal gammopathy of undetermined significance (MGUS) are characterized by an infiltration of plasma cells into the bone marrow, but the mechanisms underlying the disease progression remain poorly understood. Previous research has indicated that 3D nuclear telomeric and centromeric organization may represent important structural indicators for numerous malignancies. Here we corroborate with previously noted differences in the 3D telomeric architecture and report that modifications in the nuclear distribution of centromeres may serve as a novel structural marker with potential to distinguish MM from MGUS. Our findings improve the current characterization of the two disease stages, providing two structural indicators that may become altered in the progression of MGUS to MM.

## 1. Introduction

Multiple myeloma (MM) is an incurable malignancy characterized by an accumulation of plasma cells in the bone marrow [1]. MM is defined by the presence of end-organ damage, specifically hypercalcemia, renal failure, anemia, and bone lesions (CRAB features) [2]. Treatments are focused on the prolongation of survival, but they face numerous difficulties due to the heterogeneity of the disease, disease progression, and development of drug resistance [3]. Prolongation of survival varies from just a few months to over a decade, and conventional clinical staging is often insufficient to predict this heterogeneity in patient survival [4]. In most cases, MM develops in patients already harboring the pre-existing condition of monoclonal gammopathy of undetermined significance (MGUS), with a lack of clinical symptoms [5]. Although MGUS is a precursor of MM, the high degree of genetic and molecular heterogeneity in the plasma cells renders it difficult to distinguish those patients who are at imminent risk of progression from those that will not progress at that time [6]. Since treatment for MGUS patients before progression to MM is not yet common practice [7]; the molecular differences between disease stages could potentially distinguish the higher-risk MGUS patients.

Telomeres are structures present at the ends of chromosomes, consisting of sequential hexameric (TTAGGG)n noncoding DNA repeats and associated proteins [8]. In a normal cell, once the telomeres reach a critical length, termed the Hayflick limit, the cell will stop division and enter cell cycle arrest, or senescence [9]. By contrast, cancer cells are immortal and continue their unlimited proliferation, which has been linked in some cases to abnormal activity of telomerase and/or the Alternative Lengthening of Telomeres (ALT) pathway [10,11]. Changes in the 3D telomeric organization in the nuclear space of cancer cells have been associated with genomic instability and correlated with preneoplastic stages, disease progression, and aggressiveness [12,13,14,15,16]. In addition, genomic instability and the resulting ongoing clonal evolution have been implicated as a key driving mechanism for the heterogeneity of MM among patients [17].

Another promising approach to potentially distinguish between preneoplasia, cancer stages, and disease progression/aggressiveness is the 3D centromeric architecture. The evolutionary hotspots in the pericentromeric chromosomal regions that have been implicated as major contributors to genomic instability and large-scale genetic changes such as chromosomal rearrangements, deletions, and insertions or bursts of retrotransposon activity could force centromere positions to change [16]. However, little is known about effects of those changes in the dynamics and stability of centromeres in cancer [18,19,20]. Consequently, the positioning of the centromeres in the interphase nucleus has been the focal point of several studies (e.g., [19,20,21,22,23,24]). For instance, it has been demonstrated that the distribution of the centromeres in the nucleus of the same cells is altered in the process of immortalization and malignant transformation in both mice and humans [24,25].

Thus, our pilot study sought to investigate the spatial position and distribution of telomeres and centromeres within interphase nuclei of MM and MGUS patients, isolated from their blood or bone marrows, and determine whether 3D telomere and centromere analyses can identify patient subgroups (MGUS and MM). In this study, we aimed to investigate two structural indicators that may become altered in the progression of MGUS to MM. Our data corroborate differences in the nuclear architecture found in a previous study and highlight that modification in the distribution of centromeres can also distinguish MM from MGUS.

## 2. Materials and Methods

### 2.1. Sample Acquisition

Blood and bone marrow samples were collected at Tartu University Hospital, Tartu, Estonia from 37 patients diagnosed with MGUS and 25 patients newly diagnosed with MM. All the patients were treatment naïve and the samples were obtained within roughly a month following diagnosis. All the clinical information received is summarized in Supplementary Table S1. MGUS patients did not receive any treatment, remaining under surveillance until progression. This study was approved by the Research Ethics Review Board on Human Studies of the University of Manitoba (Ethics Reference No. H2010:170) and the Ethics Review Committee on Human Research of the University of Tartu (Protocol No. 194T-11). All research was done according to the Declaration of Helsinki.

### 2.2. Sample Processing

Patient samples were processed as previously described [23]. Blood samples and bone marrow aspirates were first overlaid in Ficoll-Paque (GE Healthcare Life Sciences, Baie d’Urfe, Quebec) and centrifuged at 200× *g* for 30 min to isolate the white blood cells. The cells were washed with 10 mL of Roswell Park Memorial Institute (RPMI) medium (Gibco Life Technologies Inc., Burlington, ON, Canada) containing 10% fetal bovine serum (FBS) (Gibco Life Technologies Inc.). Fresh slides were prepared for each sample spreading the cells onto poly-L-lysine-coated slides. The cells were fixed in a 3.7% formaldehyde solution over 20 min. The slides were then washed thrice with 1× phosphate buffered saline (PBS).

### 2.3. Immunostaining and Telomere Hybridization

For immunostaining, the cells were blocked with 4% BSA in 4X saline sodium citrate (SSC) for 15 min and incubated with Alexa Fluor^®^ 488 labeled mouse anti-human CD56 antibody (BD Biosciences, San Jose, CA, USA) and Alexa Fluor^®^ 594 labeled anti-human CD138 (Syndecan-1) (BioLegend, San Diego, CA, USA) for 60 min following three PBS washes for 5 min. To visualize the telomeres, we applied 6 μL of cyanine 3 (Cy3)-labeled peptide nucleic acid (PNA) probe (DAKO, Glostrup, Denmark) to the slides and coverslips we subsequently mounted over the cells. Probe hybridization was achieved using the HYBrite denaturation and hybridization system (Vysis; Abbott Diagnostics, Des Plaines, IL, USA) to denature the DNA at 80 °C for three minutes, before allowing the probe to hybridize to the denatured DNA at 30 °C for two hours. To wash away unbound probe, remove poor homology hybrids, and reduce signal background, the cells were washed as follows: In 70% formamide (Sigma-Aldrich, St. Louis, MO, USA)/10 mM Tris (pH 7.4) for thirty minutes, in 0.1X saline sodium citrate (SSC) at 55 °C for five minutes, and twice in 2× SSC/0.05% Tween 20 for five minutes. The nuclei were briefly counterstained with 1 µg of 4′,6-diamidino-2-phenylindole (DAPI) (Sigma-Aldrich, St. Louis, MO, USA) for 3 min, before being gently rinsed with distilled water and mounted with VECTASHIELD (Vector Laboratories, Burlington, ON, Canada).

### 2.4. Centromere Fluorescence In-Situ Hybridization (FISH)

Centromere fluorescence in-situ hybridization (FISH) was performed as previously described [25]. Briefly, the formaldehyde-fixed slides were equilibrated in 2× SSC at room temperature prior to treatment with 100 µL of 100 µg/mL RNase A solution (Thermo Fisher Scientific, Waltham, MA, USA). A ten-minute pepsin/HCl (Sigma-Aldrich, St. Louis, MO, USA) treatment at 37 °C was used to remove residual proteins, before a five-minute post-fixation was performed in 1% formaldehyde (Sigma-Aldrich, St. Louis, MO, USA). After dehydration through an ethanol series of 70%, 90%, and 100% ethanol for two minutes each (Sigma-Aldrich, St. Louis, MO, USA). The slides were prewarmed in a 70 °C oven and then denatured in 70% deionized formamide (Sigma-Aldrich, St. Louis, MO, USA) at 70 °C, before being immediately passed through an ice/cold ethanol series and hybridized with denatured fluorescein isothiocyanate (FITC)-labeled pan-centromeric probe (Cambio, Cambridge, UK) overnight. The following day, the slides were washed with 50% formamide, 2× SSC, and 4× SSC/0.1% Tween 20 before being counterstained with DAPI (1 µg for 3 min) and mounted with VECTASHIELD (Vector Laboratories, Burlington, ON, Canada) for imaging.

### 2.5. Image Acquisition

Fifty interphase nuclei were imaged per slide. The telomere and centromere-hybridized nuclei were imaged using fluorescence microscopy (Zeiss AxioImager Z1 microscope (Carl Zeiss, Toronto, ON, Canada) equipped with an AxioCam HRm camera, using a 63×/1.4 oil plan apochromat objective lens). The imaging software AXIOVISION 4.8.2 (Zeiss) and ZEN 2.3 software were used for image acquisition. 3D imaging of telomeres was performed using 80 *z*-stacks, each with a thickness of 0.2 μm (*z* plane) and 40 *z*-stacks for the centromeres. The sampling distance in both the *x*- and *y*-planes was 102 nm. The exposure time for Cy3 (telomeres) was maintained at a constant 100 milliseconds and FITC (centromeres) was held at a constant 1.1 s, whereas that for DAPI varied.

### 2.6. Identification of MM Cells

The nuclei of malignant plasma cells were differentiated from normal lymphocytes on the basis of their double staining for CD138 conjugated with Alexa Fluor^®^ 594 and CD56 conjugated with Alexa Fluor^®^ 488, augmented size, and weaker DAPI counterstain. Klewes et al. [26] confirmed that syndecan-1 (CD138) positively stained myeloma nuclei corresponded to the larger and darker nuclei. CD138 expression is specific for normal and malignant plasma cells, and CD56 expression is found in 67–79% of MM cases [27].

### 2.7. Nuclear Architecture Analysis

Fifty nuclei from each slide were deconvolved using a constrained iterative algorithm [28]. After deconvolution, the images were saved as .TIF files. The exported files were analyzed using TeloView^TM^ v1.03 software program (Telo Genomics Corp.). TeloView^TM^ shows a maximized 2D projection in the *x*-, *y*-, and *z*-planes, while performing final analyses on the original 3D images. Spots are located automatically and presented for verification and manual adjustment of the telomeres [29]. This software enabled the measurement and summarization of data including nuclear volume, telomere number, average signal intensity (proportional to telomere length), average number of aggregates (telomeres in close proximity that cannot be further resolved at an optical resolution limit of 200 nm), and spatial distribution of the telomeres within the nucleus (*a/c* ratio). These parameters measured from fifty cells were averaged and summarized for each patient. TeloView^TM^ is proprietary to Telo Genomics Corp. (Toronto, ON, Canada) and was used with the company’s permission.

For the analysis of centromeres, fifty nuclei were also analyzed, and the images were cut and then deconvolved using a constrained iterative algorithm at a strength of six for DAPI and seven for FITC in ZEN 2.3. The nuclei were then analyzed using Tools for Analysis of Nuclear Genome Organization (TANGO), an ImageJ plugin that defined the nuclei and then discerned and quantified the distance of each centromere with respect to the nuclear center [30]. This automated selection process was reviewed manually, and the data were then exported for further analysis.

### 2.8. Statistical Analyses

For statistical analysis, the software package SAS X version 9.4 (Statistical Analysis System (SAS) Institute Inc., Cary, NC, USA) was employed in performing nested factorial analysis of variance in the aforementioned parameters measured in TeloView^TM^. Chi-squared tests were used to compare the percentage of interphase telomere signals at each given intensity level at intervals of 1000 intensity units, ultimately divided into quartiles for analysis. Nested factorial analysis of variance was also used to compare the distribution of signal intensities across the two disease stages. The data obtained from TANGO were also analyzed using nested factorial analyses that compared the radial distance of the centromeres from the center of the nucleus in the two different disease stages. A *p*-value of < 0.05 was considered significant.

## 3. Results

**Figure 1:**
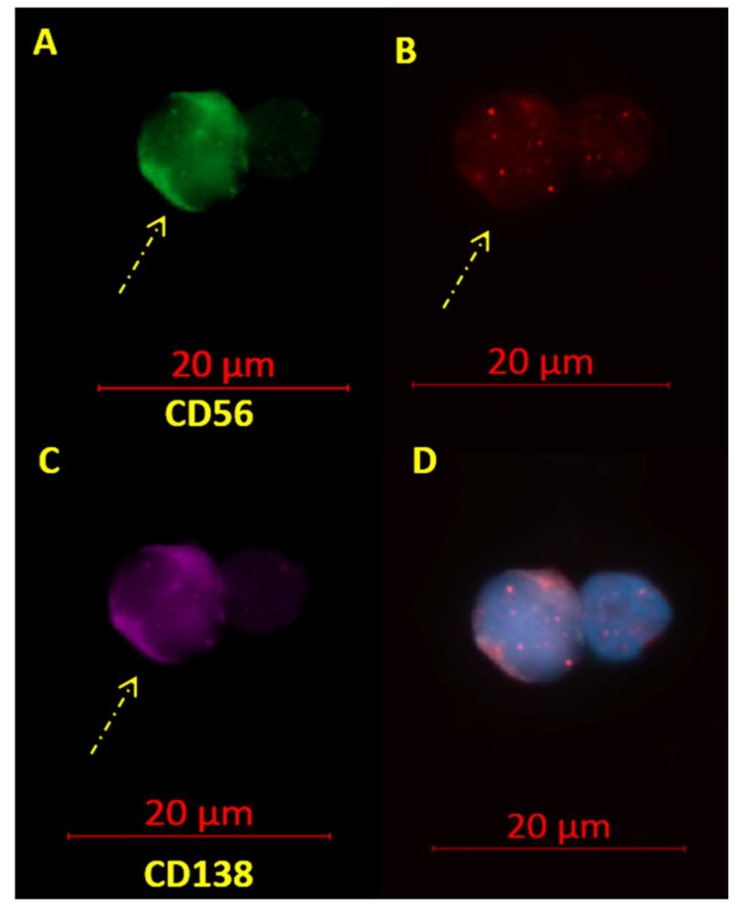
Telomere signals in CD56+ and CD138+ malignant plasma cells (**A**–**D**). (**A**) CD56+ myeloma cells fluoresce green with Alexa Fluor^®^ 488 labeled anti-CD56 antibody (see arrows) and normal cells remain unstained. (**B**) The telomeres, hybridized with cyanine 3 (Cy3)-labeled peptide nucleic acid (PNA) probes, appear as red signals. (**C**) CD138+ myeloma cells stain purple with Alexa Fluor^®^ 594 labeled anti-CD138 antibody (see arrows) while normal cells remained unstained. (**D**) Merged image with the nuclei counterstained with DAPI (blue): Myeloma cells can be differentiated from normal lymphocytes and nonmalignant plasma cells by their doubly positive CD56 and CD138 staining.

Genomic instability and disease progression can both be correlated with changes in the nuclear architecture [31]. To assess the potential of 3D telomeric and centromeric architecture as a reliable tool to differentiate MM from the precursor stage of MGUS, we evaluated a total of 62 patients (25 MM and 37 MGUS). For some patients, bone marrow samples were analyzed, while for others the samples were derived from peripheral blood, since previous comparisons had shown that identical telomeric profiles can be obtained from blood or bone marrow samples [26]. We selected the cells based on their dual positive CD56 and CD138 staining, and by their weaker DAPI counterstain and larger size; telomeres were visualized as red dots (Figure 1). Basic clinical data for the patients are summarized below (Table 1). The average age of the study population was 67 years and 72.1 years for MGUS and MM, respectively. The average percentage of plasma cell bone marrow infiltration was 6.8% and 63% for MGUS and MM, respectively, and the average amount of serum myeloma protein (M-protein) was 8.1 and 40.4 g/L for MGUS and MM, respectively. In both groups, the majority of patients had the immunoglobulin G isotype (IgG), a small proportion of both groups had the IgA isotype, and a small fraction of MGUS patients belonged to the IgM subtype. MM patients display bone marrow infiltration as well as a greatly elevated level of serum M-protein compared to MGUS patients. The most common immunoglobulin isotype in MM patients is IgG, followed by IgA [32].

From cytogenetic FISH analyses, only one MGUS patient had any of the chromosomal aberrations commonly associated with MM. However, cytogenetic data were only available for eight of the MGUS patients. Of the 25 MM patients, 19 had cytogenetic/FISH data available. Of the analyzed MM patients, 42.1% percent had a t(11;14) translocation, 21.1% a t(4;14) translocation, and 26.3% a 14q13 deletion.

**Figure 2:**
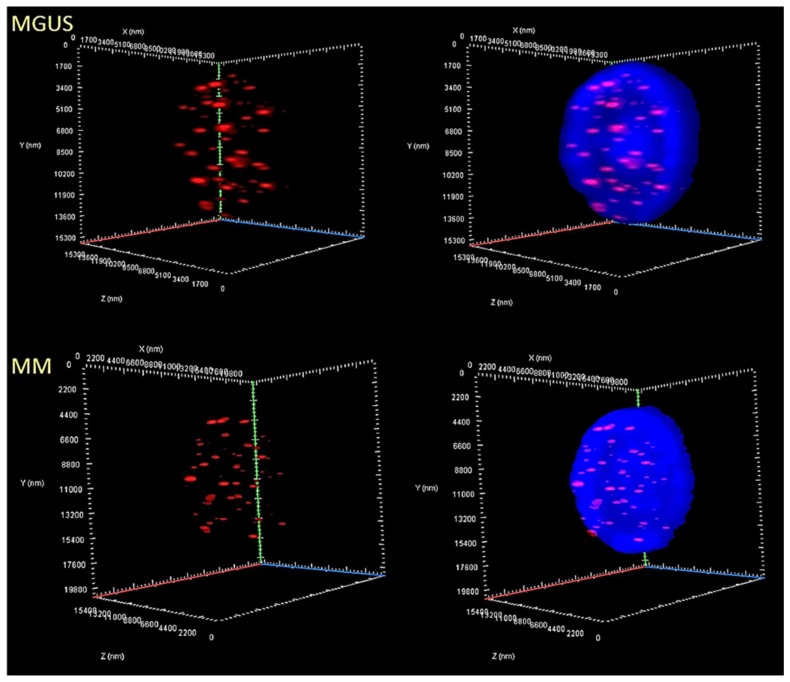
Differences in the 3D nuclear telomeric architecture between MGUS and MM. Representative nuclei, counterstained with DAPI (blue) from MGUS and MM patient samples, where Cy3 labeled telomeres appear as red dots. Numerous parameters were altered between the two disease stages. Most notably, in MM, compared to MGUS, there was a greater frequency of less-intense signals, correlating to a predominance of shorter telomeres, which by itself is indicative of telomere attrition and genomic instability.

We first assessed the total number of telomeric signals and formation of telomere aggregates using the TeloView^TM^ program [32]. There was no significant difference in the total number of signals or in the total number of telomere aggregates (*p* > 0.05) between the disease stages of MM and MGUS. We then evaluated the differences in the total and average intensity of telomeric signals (a measure of telomere length) and the nuclear volume. There was indeed a significant decrease in the average signal intensity (*p* = 0.0019) in MM compared to MGUS, indicative of a reduction in average telomere length (Figure 2), but we found no significant difference in the total intensity and nuclear volume (*p* > 0.05). In addition, we found that the number of the telomeres (number of telomeres per 1000 μm^3^ of nuclear volume) increased in MM compared to MGUS (*p* = 0.0493), pointing to the presence of more chromosomes and/or an increase in interstitial telomeres in the nuclear space (Figure 2). Table 2 summarizes these findings in the telomere architecture.

Moreover, within the nucleus, the spatial organization of the telomeres can be described using the *a/c* ratio: If the ratio is low (close to one) then it can be concluded that the telomeres are arranged in a more spherical shape, which is characteristics of the G_1_/S phase. When the *a/c* ratio is more than one, telomeres are arranged in a disk-like organization, characteristic of the G_2_ phase. A high *a/c* ratio is indicative of a greater proportion of proliferating cells and is thus a hallmark of numerous malignancies [29,33]. In the present study, we found a borderline decrease in the *a/c* ratio in MM, which did not achieve significance (*p* = 0.0597).

**Figure 3:**
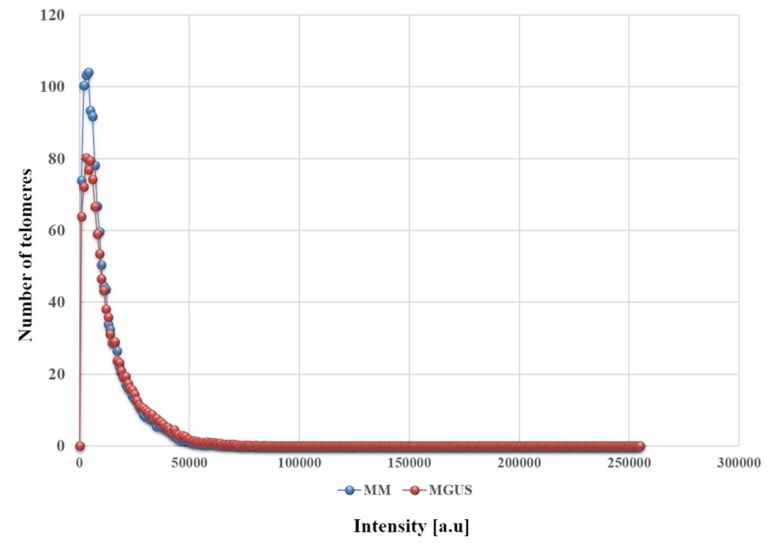
The differing frequency distributions for the intensity of telomeric signals in MGUS (red) and MM (blue). The two stages of the disease are marked by different profiles concerning the signal intensity and the number of telomeres, especially in the low-intensity and high-intensity ranges (*p* = 0.0062).

Next, we divided the telomeric signals into four quartiles based on their intensity, creating frequency distributions based on the frequency of signals with each particular intensity. We found that the frequency distributions of the two disease stages was significantly different between MM and MGUS (*p* = 0.0062). Interestingly, this difference seems to be unrelated to any difference in the lowest-intensity signals (*p* = 01873), but rather associated with a greater frequency of the highest-intensity signals (>46,000 arbitrary units) in MGUS (*p* = 0.0005) in our quartile analysis. In Figure 3, telomere length (signal intensity) (*x*-axis) is plotted against the number of telomeres (*y*-axis) for all cells analyzed (for each patient). Signals are grouped by their intensity level and this gives a picture of the telomere distribution in each sample or time point. For normal lymphocytes, for example, this plot usually has small peaks between 0 and 20,000 a.u. (relative fluorescence intensity), in which the number of telomeres per nucleus on the *y*-axis range between 5 and 25 in this bin. In normal lymphocytes, most of the telomere signals have high relative intensities, with signals detected in each bin up to 120,000 a.u. [16]. Thus, MM could be defined by an overall enrichment in shorter telomeres and an absence of very long telomeres compared to MGUS as reflected in the profiles of the frequency distributions (Figure 3).

**Figure 4:**
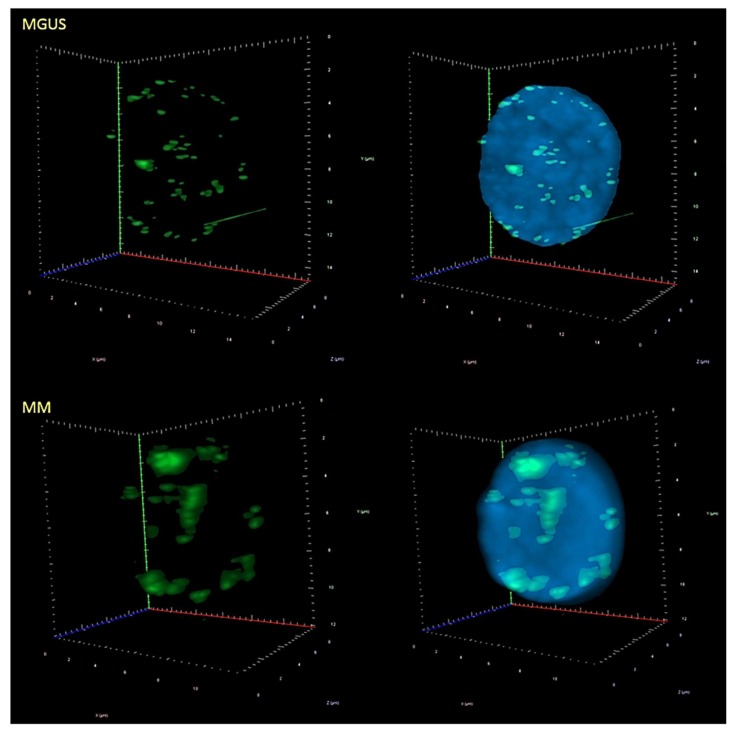
Three-dimensional (3D) images of representative nuclei from MGUS and MM patient samples showing the centromere distribution patterns. The FITC-labeled pan-centromeric probe in green indicates the centromeres in the cells, while the nuclei are counterstained in blue with DAPI. In MGUS samples, the centromeres generally occur as small punctate signals, but in MM samples they often form indistinct clusters.

**Figure 5:**
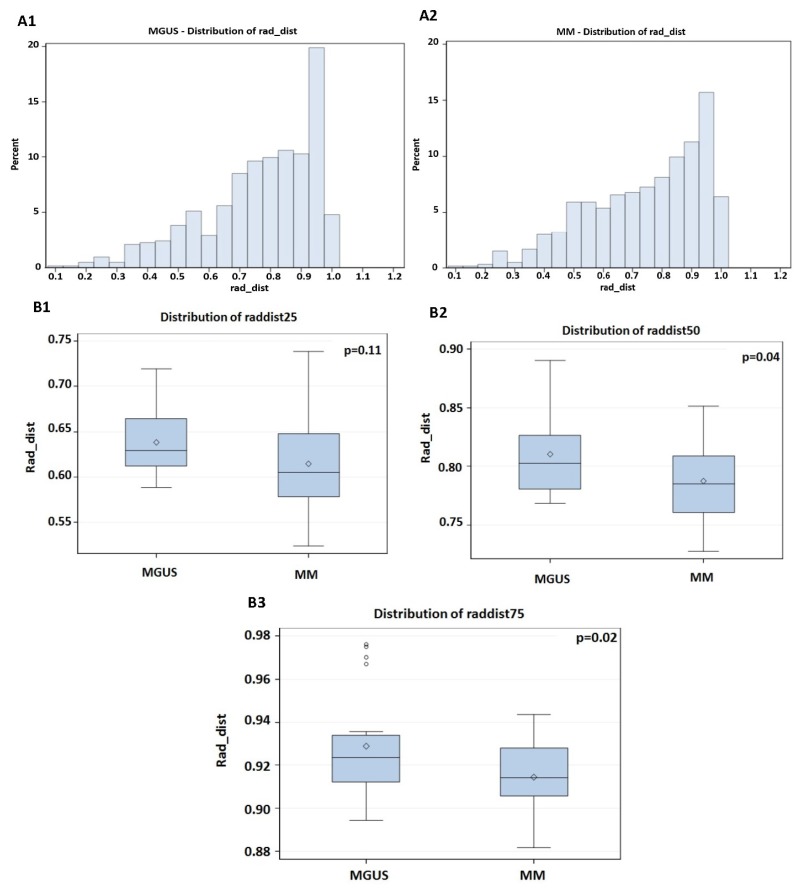
Histograms of the centromeric frequency distributions along a radial nuclear axis starting from the nuclear center (0) to the nuclear periphery (1.0) for all MGUS and MM patient groups (**A1** and **A2**). In **B1**–**B3**, the differences in centromeric distribution frequencies are represented. Centromeric signals are divided into four quartiles by groups MGUS and MM. The rad_dist are ranked from lowest to highest, and where 25% of the distance from lowest is named raddist25 (the q1—first quartile) (close to the nucleus) (**B1**), where 50% (half) is named raddist50 (this is also referred to as the median—midpoint between the nuclear center and the nuclear periphery) (**B2**), and where 75% is named raddist75 (third quartile) (close to the periphery of the cell) (**B3**). Centromeres in cells from MGUS patients displayed a significantly greater distribution in both subdivisions (midpoint between the nuclear center and the nuclear periphery and close to the periphery of the cell) of this region than in those from MM patients, indicating that the centromeres are more distributed toward the nuclear periphery than the nuclear center in MGUS than in MM. The *p*-values for the comparison between MGUS and MM are included in each graph.

We next proceeded to assess the differences in centromeric organization, for the same patient cohort (62 patients—25 MM and 37 MGUS), using TANGO [30]. TANGO detects centromeric signals and determines their 3D spatial position within the nucleus, enabling the detection of differences in the signal distribution between different disease stages (Figure 4). The radial distance between the region and the nuclear center is fractionally expressed in terms of the measured distance of this radial arm, with 0 being at the nuclear center and 1 representing the nuclear periphery. The centromeric signals were divided into four quartiles per patient: The radial distances were ranked from lowest to highest—where raddist25, raddist50, and raddist75 represent 25%, 50%, and 75% of the distances from lowest, respectively. Raddist25 is also called q1 or the first quartile, raddist50 is also referred to as the median or half, and raddist75 is the third quartile. As seen in Figure 5, MGUS cells had a higher frequency of centromeres than MM cells within the more peripheral nuclear radial space of 0.5–0.75, and 0.75–1 (*p* = 0.04 and *p* = 0.02). In the first two quartiles, i.e., within the nuclear radial space from 0 to 0.5, which is closer to the center of the nucleus, both MGUS and MM cells had a similar frequency distribution of centromeres.

## 4. Discussion

This study reports on the evaluation of blood and bone marrow samples from a cohort of 62 patients diagnosed with MGUS (37) and MM (25), in order to assess the usefulness of telomeres and centromeres as reliable tools for stratifying patients into subgroups based on their 3D nuclear organization that is indicative of their levels of genomic instability. We found several significant differences in the nuclear architecture of the two disease stages, corroborating with some of the trends observed by Klewes et al. 2013 [26], such as a decrease in the average telomere signal intensity, confirming that an increase in very short telomeres is a common phenomenon in MM and a decrease in the *a/c* ratio from MGUS to MM. The overall, shorter telomere length in MM compared to MGUS patient samples has also been described by Hyatt et al. 2017 [34], as well as by Wu et al. 2003 [35] and Cottliar et al. 2003 [36]. However, Hyatt et al. [34] used a genomic DNA PCR-based method known as single telomere length analysis (STELA) with a pool of cells, whereas the latter two studies employed terminal restriction fragment (TRF) analysis via Southern blotting. Q-FISH presents several advantages over these methods due to its greater ease of use [37] and its single-cell analyses. Single cell analyses with Q-FISH not only require less overall patient material, but also allow for better assessment of clonal diversity and the variability inherent to a cell population, enabling early detection of clones that may be treatment resistant, metastatic, or indicative of clonal evolution and aggressiveness [38]. Moreover, the 3D nature of these analyses enables a visual and quantitative interrogation of parameters not possible through other methods, for instance the proximity of telomeres to one another and the formation of telomere aggregates, as well as the 3D spatial distribution of structures such as the telomeres and centromeres in the nuclear space, in turn allowing for investigation of the nuclear 3D organization in relation to disease [39].

One such parameter is the *a/c* ratio (the nuclear space occupied by telomeres and represented by three axes of length *a, b*, and *c*), which is dynamic, and changes at different stages of the cell cycle (First and second gap phases, and synthesis stage: G_0_/G_1_, S, G_2_). Two previous studies from our group had demonstrated the concordance between the *a/c* ratio and the cell cycle phase [29,33]. As cells progress through the cell cycle, the *a/c* ratio remains relatively unchanged even through DNA synthesis in the S phase, but it increases drastically for cells entering the G_2_ phase, which corresponds to the telomeres becoming organized in a disk-like formation in preparation for mitosis. At this point, the *a/c* ratio is typically around 14 ± 2, rapidly decreasing to one again with the passing of mitosis [29]. The *a/c* ratio was not statistically significant between the MGUS (*a/c* ratio of 5.2) and MM (*a/c* ratio of 4.5) patient cohorts. One plausible explanation is that our samples were derived both from bone marrow and peripheral blood, whereas most studies showing differences in the proliferation rates only analyzed cells from their main environment, the bone marrow. 

On the other hand, investigations into the 3D centromeric architecture of the cell have been lacking in the literature. This is, to the best of our knowledge, the first report demonstrating the differences in the 3D distribution of centromeres throughout the nucleus between MM and MGUS. Sarkar et al. [25] previously established that, in mice, centromeres are located more toward the nuclear periphery in normal lymphocytes but are more concentrated in the middle of the nucleus in immortalized PreB lymphocytes and even more so in the murine plasmacytoma line MOPC460D [25]. Wark et al. [20] showed that the spatial position of centromeres in interphase nuclei was altered in fibroblast cell lines with monoallelic truncating *PALB2* mutations (PALB2 c.3323delA) compared to wild-type controls and fibroblasts with *BRCA1* and *BRCA2* mutations. This difference was found to be a contributing factor in increasing the propensity for chromosomal rearrangements [20]. We found centromeres in all locations of the nucleus in MGUS and MM patient samples. However, the significant differences between groups were found in centromeres displaying a radial distance between 0.5 and 1, i.e., located in the midpoint between the nuclear center and the nuclear periphery (a) and the nuclear periphery itself (b). MGUS patients have more centromeres that are farther away from the two subdivisions measured (a and b) (*p* = 0.04 and *p* = 0.02). Interestingly, we also observed that the centromeres in MM patient samples form clusters, unlike those from MGUS cells (Figure 4). This observation indicates that chromosome positions have been altered and/or that some chromosomes (more likely the acrocentric ones) may have broken, rearranged, or fused [16].

Our results suggest that the differences in centromeric distribution and telomeric architecture point to nuclear remodeling as an important feature of the transformation from MGUS to MM. Such differences are also observed between normal lymphocytes and malignant cells for both telomeric length distribution profiles [29] and centromeric distribution in the nucleus [25]. We propose that these differences in the 3D nuclear architecture reflect an altered nuclear organization during malignant transformation, with potential implications for genomic instability and DNA damage. Previous studies have investigated whether primary malignant plasma cells from patients with MM or MGUS present with phosphorylated H2AX, as evidence of DNA damage [40]. Indeed, this was found to be the case for 90% of the MM patients and MM cell lines but γH2AX foci formation was only detected in a few of the MGUS patients. However, in the same study, they included both untreated and treated MM patients, which likely affected their results. While there are no data for myeloma linking γH2AX foci formation with telomere shortening, in breast cancer patients, γ-H2AX foci were linked to shorter telomeres, which were in turn associated with poorer prognosis of triple-negative breast cancer patients [41]. As such, future studies are necessary to fully understand the implications of these alterations to the 3D nuclear architecture for MM prognosis.

One aspect not included in our study is an exploration of smoldering multiple myeloma (SMM). Smoldering myeloma represents a precursor stage with a higher degree of M-protein secretion and bone marrow plasma cell infiltration than MGUS [42]. SMM is of pressing importance because patients with the condition develop MM at an augmented rate of 10% per annum, as opposed to 1% per annum for MGUS patients [43]. The nuclear architecture of SMM patients should be assessed to establish whether differences exist that could not only help distinguish it from other stages but also shed light on the relation between telomere and centromere architecture and disease progression.

New studies are needed in order to elucidate what factors in the nuclear architecture may predict the transformation process—why most MGUS patients will remain stable while some develop SMM or even full-blown MM is still poorly understood at the molecular level. Previous research has shown that nuclear architecture can be a useful tool in further stratifying patients with many cancers and heterogeneous diseases such as glioblastomas [33], neuroblastomas [44], and plasmacytomas [45]. There is a great likelihood that further research will allow the creation of more specifically targeted, personalized treatments and improved diagnostic methods to optimize patient care and outlook for those afflicted by MM. Indeed, a recent publication by Kumar et al. 2018 reinforced the role of telomeres for developing therapies, linking treatment of myeloma cell lines with tanshinone I and lenalidomide to a reduction in telomerase activity and proteins associated with telomere protection [46].

Here we corroborate previously noted differences in the 3D telomeric architecture and report that modifications in the nuclear distribution of centromeres may serve as a second structural marker with potential to distinguish MM from MGUS. Our findings thus improve upon the current characterization of the two disease stages.

## Figures and Tables

**Table 1:** Basic clinical characteristics of the Estonian study population assessed at time of diagnosis.

	MGUS	MM
Sample Number (*n*)	37	25
Basic Clinical Characteristics		
Age	67.0 ± 18	72.1 ± 35.1
BMPC (%)	6.8 ± 13.2	63.0 ± 48.0
M-protein (g/L)	8.1 ± 5.6	40.4 ± 38.6
IgG (%)	59.5	52.0
IgA (%)	16.2	12.0
IgM (%)	5.4	0
n.d. (%)	18.9	36.0
Cytogenetics Information		
Patients with t(11;14) (%)	1/8 (12.5%)	8/19 (42.1%)
Patients with t(4;14) (%)	0/8	4/19 (21.1%)
Patients with del(14q13)/13qter (%)	0/8	5/19 (26.3%)
Patients lacking cytogenetics information	29	6

BMPC indicates the degree of bone marrow plasma cell infiltration; M-protein indicates the serum level of myeloma protein. IgG, IgA, and IgM indicate the percentage of patients in each cohort with each of the three isotypes of immunoglobulin heavy chain as the predominant isotype; n.d. is the percentage of patients whose immunoglobulin isotype data were unavailable. Numbers represent average values along with the maximal variance in these values.

**Table 2:** Summary of parameters of 3D nuclear telomere architecture, according to monoclonal gammopathy of undetermined significance (MGUS) or multiple myeloma (MM) diagnosis (significant results are marked with an asterisk; a.u.—arbitrary units). *p*-value of < 0.05 was considered significant.

		Nuclear 3D Telomere Parameters						
Diagnosis	Number of Subjects	Number of Telomeres	Number of Telomere Aggregates	a/c Ratio	Average Intensity of Signals (a.u.)	Total Intensity of Signals (a.u.)	Nuclear Volume (µm^3^)	Telomeres per 1000 µm^3^
MGUS	37	42.3813	4.5800	5.1972	13,604.53	568,153.17	415,857.81	0.1147
MM	25	45.3356	5.0719	4.4858	12,298.72	540,375.19	436,850.64	0.1380
Differences		*p* = 0.1096	*p* = 0.1344	*p* = 0.0597	*p* = 0.0019*	*p* = 0.2604	*p* = 0.6521	*p* = 0.0493*

## Supplementary Materials

The following are available online at https://www.mdpi.com/2073-4409/8/7/723/s1, Table S1: Clinical data for participating patients.

## References

[1] Kazandjian D. (2016). Multiple myeloma epidemiology and survival: A unique malignancy. Semin. Oncol..

[2] Anderson K.C. (2011). Multiple myeloma: A clinical overview. Oncol. Williston Park.

[3] San-Miguel J.F., Mateos M.-V. (2011). Can multiple myeloma become a curable disease?. Haematologica.

[4] Kumar S.K., Dispenzieri A., Lacy M.Q., Gertz M.A., Buadi F.K., Pandey S., Rajkumar S.V. (2014). Continued improvement in survival in multiple myeloma: Changes in early mortality and outcomes in older patients. Leukemia.

[5] Landgren O., Kyle R.A., Pfeiffer R.M., Katzmann J.A., Caporaso N.E., Hayes R.B., Hoover R. (2009). Monoclonal gammopathy of undetermined significance (MGUS) consistently precedes multiple myeloma: A prospective study. Blood.

[6] Corre J., Munshi N., Avet-Loiseau H. (2015). Genetics of multiple myeloma: Another heterogeneity level?. Blood.

[7] Van Nieuwenhuijzen N., Spaan I., Raymakers R., Peperzak V. (2018). From MGUS to multiple myeloma, a paradigm for clonal evolution of premalignant cells. Cancer Res..

[8] Shay J.W., Wright W.E. (2011). Role of telomeres and telomerase in cancer. Semin. Cancer Biol..

[9] Shay J.W., Wright W.E. (2000). Hayflick, his limit, and cellular ageing. Nat. Rev. Mol. Cell Biol..

[10] Svenson U., Roos G. (2011). Telomere length as a biological marker in malignancy. Biochim. Biophys. Acta.

[11] Shiratuschi M., Muta K., Abe Y., Motomura S., Taguchi F., Takatsuki H., Nishimura J. (2002). Clinical significance of telomerase activity in multiple myeloma. Cancer.

[12] Nabetani A., Ishikawa F. (2011). Alternative lengthening of telomeres pathway: Recombination-mediated telomere maintenance mechanism in human cells. J. Biochem..

[13] Saito T., Hama S., Izumi H., Yamasaki F., Kajiwara Y., Matsuura S., Kurisu K. (2008). Centrosome amplification induced by survivin suppression enhances both chromosome instability and radiosensitivity in glioma cells. Br. J. Cancer.

[14] Mai S., Garini Y. (2006). The significance of telomeric aggregates in the interphase nuclei of tumor cells. J. Cell. Biochem..

[15] Rangel-Pozzo A., de Souza D.C., Schmid-Braz A.T., Azambuja A.P., Ferraz-Aguiar T., Borgonovo T., Mai S. (2019). 3D Telomere Structure Analysis to Detect Genomic Instability and Cytogenetic Evolution in Myelodysplastic Syndromes. Cells.

[16] Awe J.A., Xu M.C., Wechsler J., Benali-Furet N., Cayre Y.E., Saranchuk J., Mai S. (2013). Three-dimensional telomeric analysis of isolated circulating tumor cells (CTCs) defines CTC subpopulations. Transl. Oncol..

[17] Cagnetta A., Lovera D., Grasso R., Colombo N., Canepa L., Ballerini F., Cea M. (2015). Mechanisms and clinical applications of genome instability in multiple myeloma. BioMed. Res. Int..

[18] Malik H.S., Henikoff S. (2009). Major evolutionary transitions in centromere complexity. Cell.

[19] Gonçalves dos Santos Silva A., Sarkar R., Harizanova J., Guffei A., Mowat M., Garini Y., Mai S. (2008). Centromeres in cell division, evolution, nuclear organization and disease. J. Cell. Biochem..

[20] Wark L., Novak D., Sabbaghian N., Amrein L., Jangamreddy J.R., Cheang M., Tischkowitz M. (2013). Heterozygous mutations in the PALB2 hereditary breast cancer predisposition gene impact on the three-dimensional nuclear organization of patient-derived cell lines. Genes Chromosomes Cancer.

[21] McKinley K.L., Cheeseman I.M. (2016). The molecular basis for centromere identity and function. Nat. Rev. Mol. Cell Biol..

[22] Weierich C., Brero A., Stein S., von Hase J., Cremer C., Cremer T., Solovei I. (2003). Three-dimensional arrangements of centromeres and telomeres in nuclei of human and murine lymphocytes. Chromosome Res..

[23] Ollion J., Loll F., Cochennec J., Boudier T., Escudé C. (2015). Proliferation-dependent positioning of individual centromeres in the interphase nucleus of human lymphoblastoid cell lines. Mol. Biol. Cell.

[24] Voldgorn Y.I., Adilgereeva E.P., Nekrasov E.D., Lavrov A.V. (2015). Cultivation and differentiation change nuclear localization of chromosome centromeres in human mesenchymal stem cells. PLoS ONE.

[25] Sarkar R., Guffei A., Vermolen B.J., Garini Y., Mai S. (2007). Alterations of centromere positions in nuclei of immortalized and malignant mouse lymphocytes. Cytom. Part A.

[26] Klewes L., Vallente R., Dupas E., Brand C., Grün D., Guffei A., Mai S. (2013). Three-dimensional nuclear telomere organization in multiple myeloma. Transl. Oncol..

[27] Van Camp B., Durie B.G., Spier C., De Waele M., Van Riet I., Vela E., Grogan T.M. (1990). Plasma cells in multiple myeloma express a natural killer cell-associated antigen: CD56 (NKH-1; Leu-19). Blood.

[28] Schaefer L.H., Schuster D., Herz H. (2001). Generalized approach for accelerated maximum likelihood based image restoration applied to three-dimensional fluorescence microscopy. J. Microsc..

[29] Vermolen B.J., Garini Y., Mai S., Mougey V., Fest T., Chuang T.C.-Y., Young I.T. (2005). Characterizing the three-dimensional organization of telomeres. Cytom. Part A.

[30] Ollion J., Cochennec J., Loll F., Escude C., Boudier T. (2013). TANGO: A generic tool for high-throughput 3D image analysis for studying nuclear organization. Bioinformatics.

[31] Mai S., Garini Y. (2005). Oncogenic remodeling of the three-dimensional organization of the interphase nucleus. Cell Cycle.

[32] González D., van der Burg M., García-Sanz R., Fenton J.A., Langerak A.W., González M., Morgan G.J. (2007). Immunoglobulin gene rearrangements and the pathogenesis of multiple myeloma. Blood.

[33] Gadji M., Fortin D., Tsanaclis A.M., Garini Y., Katzir N., Wienburg Y., Mai S. (2010). Three-dimensional nuclear telomere architecture is associated with differential time to progression and overall survival in glioblastoma patients. Neoplasia.

[34] Hyatt S., Jones R.E., Heppel N.H., Grimstead J.W., Fegan C., Jackson G.H., Baird D.M. (2011). Telomere length is a critical determinant for survival in multiple myeloma. Br. J. Haematol..

[35] Wu K.D., Orme L.M., Shaughnessy J., Jacobson J., Barlogie B., Moore M.A. (2003). Telomerase and telomere length in multiple myeloma: Correlations with disease heterogeneity, cytogenetic status, and overall survival. Blood.

[36] Cottliar A., Pedrazzini E., Corrado C., Engelberger M.I., Narbaitz M., Slavutsky I. (2003). Telomere shortening in patients with plasma cell disorders. Eur. J. Haematol..

[37] O’Sullivan J.N., Finley J.C., Risques R.A., Shen W.T., Gollahon K.A., Moskovitz A.H., Rabinovitch P.S. (2004). Telomere length assessment in tissue sections by quantitative FISH: Image analysis algorithms. Cytom. Part A.

[38] Tellez-Gabriel M., Ory B., Lamoureux F., Heymann M.F., Heymann D. (2016). Tumour heterogeneity: The key advantages of single-cell analysis. Int. J. Mol. Sci..

[39] Mogilner A., Odde D. (2011). Modeling cellular processes in 3D. Trends Cell Biol..

[40] Walters D.K., Wu X., Tschumper R.C., Arendt B.K., Huddleston P.M., Henderson K.J., Dispenzieri A., Jelinek D.F. (2011). Evidence for ongoing DNA damage in multiple myeloma cells as revealed by constitutive phosphorylation of H2AX. Leukemia.

[41] Nagelkerke A., Van Kuijk S.J., Martens J.W., Sweep F.C., Hoogerbrugge N., Bussink J., Span P.N. (2015). Poor prognosis of constitutive γ-H2AX expressing triple-negative breast cancers is associated with telomere length. Biomark. Med..

[42] Kim J.H., Kim J.W., Kim Y.N., Kim H.I., Kim J.Y., Kwon G.Y., Jang H.R. (2015). Progression of monoclonal gammopathy with undetermined significance to multiple myeloma diagnosed by kidney biopsy: A case report. Case Rep. Nephrol. Dial..

[43] Kyle R.A., Thurneau T.M., Rajkumar S.V., Offord J.R., Larson D.R., Plevak M.F., Melton L.J. (2002). A long-term study of prognosis in monoclonal gammopathy of undetermined significance. N. Engl. J. Med..

[44] Kuzyk A., Gartner J., Mai S. (2016). Identification of neuroblastoma subgroups based on three-dimensional telomere organization. Transl. Oncol..

[45] Kuzyk A., Mai S. (2012). Selected telomere length changes and aberrant three-dimensional nuclear telomere organization during fast-onset mouse plasmacytomas. Neoplasia.

[46] Kumar R., Gupta N., Himani, Sharma A. (2018). Novel combination of tanshinone I and lenalidomide induces chemosensitivity in myeloma cells by modulating telomerase activity and expression of shelterin complex and its associated molecules. Mol. Biol. Rep..

