# Supplementary material for: Distinct Nuclear Organization of Telomeres and Centromeres in Monoclonal Gammopathy of Undetermined Significance and Multiple Myeloma
Source: Cells. 2019 Jul 15;8(7):723. doi: 10.3390/cells8070723 (PMC6678424; doi:10.3390/cells8070723)
Supplement: Supplementary file 1 [file cells-08-00723-s001.pdf]

Supplemental Table S1. Clinical data for participating patients.

							Light Chain Isotype		Serum Free Light Chain								
Patient Sample	Age	Sampled Material	BMPC %	IgG	IgA	IgM	Kappa	Lambda	Kappa	Lambda	Ratio	M Band g/l	Lytic Lesions	t(11;14)	t(4;14)	del13q14 /13qter	CD138^{+}/ CD56^{+*}
MGUS1TRT	82	PB	10	5.7	0.57	6.58			35.08	11.81	2.97	3.4	no	N/A	N/A	N/A	0.38%
MGUS3TRT	75	PB	10-20	6.7	5.13	0.61		Yes	10.54	203.69	0.05	2.1	no	N/A	N/A	N/A	0.47%
MGUS2TRT	52	PB	7-10	12.8	2.34	1.59	Yes		31.06	21.06	1.47	4.3	no	N/A	N/A	N/A	0.56%
MGUS4TRT	50	PB	N/A	8.9	1.96	6.99	Yes		2.31	1.19	1.94	6	no	N/A	N/A	N/A	0.46%
MGUS5TRT	72	PB	20	19.3	0.73	0.38		Yes	13.51	15.32	0.88	9.4	no	N/A	N/A	N/A	0.56%
MGUS6TRT	84	PB	N/A	16.9	1.71	0.96	Yes		21.33	21.82	0.97	7.4	no	N/A	N/A	N/A	0.37%
MGUS7TRT	64	PB	5	16.3	2.26	0.89	Yes		25.47	15.93	1.6	4.8	no	N/A	N/A	N/A	0.47%
MGUS9.1TRT	53	PB	5	14.9	2.16	1.71		Yes	10.49	12.51	0.84	5.4	no	N/A	N/A	N/A	0.55%
MGUS10TRT	56	PB	10	13.6	0.49	0.39		Yes	0.08	7.56	91.37	7.9	no	No	No	No	0.48%
MGUS11TRT	69	PB	5	10.4	9.53	1.39	Yes		28.3	24.13	1.17	5	no	N/A	N/A	N/A	0.58%
MGUS12TRT	73	PB	10	17	1.34	1.55	Yes		44.04	12.06	3.65	8.6	no	N/A	N/A	N/A	0.48%
MGUS13TRT	74	BM	0	15.1	1.5	4			N/A	N/A	N/A	7	no	N/A	N/A	N/A	0.59%
MGUS14TRT	71	PB	7-10	27.1	1.23	1.08	Yes		6.39	1.58	4.04	16	no	N/A	N/A	N/A	0,43%
MGUS15TRT	73	PB	N/A	14.6	2.18	1.15			18.13	13.32	1.36	6.2	no	N/A	N/A	N/A	0.48%
MGUS16TRT	69	PB	<5	20	3.5	0.75	Yes		3.65	3.72	0.98	13.6	no	N/A	N/A	N/A	0.55%
MGUS17TRT	64	PB	N/A	11.3	1.49	0.63			12.55	13.58	0.92	5.2	no	N/A	N/A	N/A	0.48%
MGUS18TRT	69	PB	7	15	2.24	0.53		Yes	26.24	45.53	0.58	4.7	no	N/A	N/A	N/A	0.56%
MGUS19TRT	67	PB	N/A	15.7	0.23	0.24	Yes		10.58	6.27	1.69	13.4	no	N/A	N/A	N/A	0.57%
MGUS21TRT	75	PB	10	22.6	0.84	0.56			13.58	9.87	1.38	15.8	yes	No	No	No	0.65%
MGUS22TRT	74	PB	7–10	15.3	1.69	0.31			22.02	33.92	0.65	7	no	N/A	N/A	N/A	0.56%
MGUS23TRT	63	PB	N/A	13.7	2	1.05		Yes	18.5	26.68	0.69	7.5	no	N/A	N/A	N/A	0.46%
MGUS24TRT	68	BM	4	9	2.8	1.3		Yes	11.13	11.58	0.96	3.5	no	No	No	No	0.65%
MGUS25TRT	83	BM	<5	16.8	1.38	0.71		Yes	17.16	21.47	0.8	13.7	no	No	No	No	0.56%
MGUS26TRT	62	PB	N/A	8.8	6	1.14		Yes	18.68	106.37	0.18	1.8	no	N/A	N/A	N/A	0.67%
MGUS27.1TRT	42	PB	N/A	8.3	1.37	1.13	Yes		11.84	116.89	0.10	3.4	no	N/A	N/A	N/A	0.65%
MGUS36TRT	68	BM	5	18	3.78	2.4	Yes		22.37	17.86	1.25	9.6	no	N/A	N/A	N/A	0.56%
MGUS37TRT	72	PB	<5	11.3	2.18	1.37	Yes		23.74	19.94	1.19	3.5	no	No	No	No	0.46%
MGUS38TRT	54	PB	10	8.5	4.74	0.96		Yes	10.6	32.37	0.33	4.1	no	N/A	N/A	N/A	0.56%

MGUS39TRT	83	PB	10	9.3	10.3	0.82		Yes	22.48	27.08	0.83	4.6	no	N/A	N/A	N/A	0.58%
MGUS40TRT	80	PB	10	9.3	10.73	0.93		Yes	N/A	N/A	N/A	7.8±2.3	no	N/A	N/A	N/A	0.79%
MGUS42TRT	69	PB	5	11.6	3.36	1.46	Yes		22.17	13.83	1.6	0	N/A	N/A	N/A	N/A	0.67%
MGUS43TRT	77	PB	3	10.8	3.1	0.85	Yes		35.67	21.95	1.63	2.7	no	N/A	N/A	N/A	0.67%
MGUS44TRT	64	PB	5	13.9	2.64	0.88	Yes		22.31	14.8	1.5	8.6	no	Yes	No	No	0.56%
MGUS45TRT	57	PB	3-4	11.8	3.15	1.32		Yes	17.48	11.8	1.32	2.2	no	No	No	No	0.55%
MGUS46TRT	61	PB	N/A	10.8	0.82	0.78			N/A	N/A	N/A	6.4	no	N/A	N/A	N/A	0.67%
MGUS49TRT	49	PB	5–10	13.6	2.08	1		Yes	24.56	21.63	1.14	6.8	no	No	No	No	0.58%
MGUS50TRT	60	PB	2	11.6	2.22	1.43	Yes		47.02	23.68	1.99	5	no	N/A	N/A	N/A	0.65%
MM2TRT	61	BM	90	3.7	33.4	<0.05		Yes	10.96	2317.75	0.00	25.5	yes	N/A	N/A	N/A	1%-
MM3TRT	82	PB	90	76	<0.15	<0.05		Yes	10.46	3092.7	0.00	48	yes	N/A	N/A	N/A	1%-
MM4TRT	78	BM	90	3	0.25	<0.05		Yes	18.18	7909	0.00	1.8	yes	No	No	No	1%-
MM5TRT	71	PB	75	11.1	0.24	0.06	Yes		N/A	N/A	N/A	5.2	yes	N/A	N/A	N/A	1%-
MM6TRT	37	PB	95	5.9	0.34	<0.05		Yes	3.68	2295	0.00	3.4	no	No	No	No	1%-
MM7TRT	85	PB	95-100	40.2	0.28	<0.05		Yes	13.78	5469	0.00	34.6	yes	No	No	No	1%-
MM8TRT	89	BM	95	61.6	<0.3	<0.05	Yes		710.34	16.01	44.37	60.3	yes	N/A	N/A	N/A	1%-
MM9TRT	75	BM	15	84.8	0.36	0.17	Yes		1529.4	13.45	113.7	42.6	yes	No	No	No	1%-
MM10TRT	76	BM	60	42.7	<0.15	0.1	Yes		297.78	12.24	24.33	35.1	yes	Yes	No	No	1%-
MM11TRT	71	BM	50	37.5	0.49	0.65			15.28	6.82	2.24	31	no	N/A	N/A	N/A	1%-
MM12TRT	52	PB	25	24	0.4	0.31	Yes		757.99	3.53	214.73	19	yes	Yes	No	No	1%-
MM13TRT	64	BM	90	66.2	0.54	0.34	Yes		288.92	5.7	50.69	74.3	yes	No	No	No	1%-
MM14TRT	87	BM	70	3.1	43.6	0.16		Yes	12.64	0.02	821.51	36.9	no	Yes	No	No	1%-
MM15TRT	74	BM	50	41.5	<0.15	0.13	Yes		691.17	3.09	223.68	29	yes	Yes	Yes	Yes	1%-
MM20TRT	70	BM	30-40	25.2	<0.15	0.12	Yes		32.52	3.82	8.51	21.9	no	Yes	No	No	1%-
MM21TRT	60	BM	60	4.8	0.6	0.42	Yes		27300	17.38	1570.77	17	yes	No	No	Yes	1%-
MM22TRT	87	BM	15	24.1	0.17	0.16	Yes		57.45	7.99	7.19	26.2	yes	N/A	N/A	N/A	1%-
MM23TRT	58	PB	25	45.4	0.42	0.65	Yes		47.07	2.59	18.17	41	yes	No	No	No	1%-
MM24TRT	68	BM	90	59.2	0.25	0.16	Yes		4470.74	7.49	596.89	79	yes	Yes	Yes	No	1%-
MM25TRT	89	BM	80	60.6	<0.15	0.37	Yes		1292.13	8.8	146.83	67.3	yes	Yes	No	No	1%-
MM29TRT	84	BM	85	45.9	<0.15	<0.15		Yes	11.7	3361.35	0.00	44.7	yes	No	No	Yes	1%-
MM30TRT	69	BM	20	7.5	0.69	0.38			95.62	10.36	9.23	0	yes	No	No	No	1%-
MM31TRT	60	BM	60	5.4	0.42	0.18		Yes	9.27	2383.24	0.00	5	yes	No	No	No	1%-
MM37TRT	68	BM	80	5.9	0.85	0.19	Yes		6781.8	6.1	1111.77	2.7	yes	Yes	Yes	Yes	1%-

MM38TRT	88	BM	40	5.1	16.1	0.24		Yes	9.98	92.21	0.11	11.8	no	No	No	1%-

Legend: PB – peripheral blood; BM – Bone marrow; * - percentage of $CD138^+/CD56^+$ positive cells found in 10^5 isolated cells using Ficoll-Paque; 1%- More than 1% of cells $CD138^+/CD56^+$ positive.
